# Supply chains and delivery systems for pig vaccines in Uganda – Challenges and solutions

**DOI:** 10.4102/ojvr.v93i1.2246

**Published:** 2026-06-10

**Authors:** Emmanuel Hasahya, Peter Oba, Emily A. Ouma, Rebecca Doyle, Theodore J.D. Knight-Jones, Michel Dione

**Affiliations:** 1 International Livestock Research Institute, Kampala, Uganda; 2 Department of People, Policies and Institutions, International Livestock Research Institute, Kampala, Uganda; 3 Royal (Dick) School of Veterinary Studies, University of Edinburgh, Edinburgh, United Kingdom; 4 Animal and Human Health Program, International Livestock Research Institute, Addis Ababa, Ethiopia; 5 Animal and Human Health Program, International Livestock Research Institute, Bamako, Mali

**Keywords:** supply chain, pigs, vaccines, value chain actors, Uganda

## Abstract

Pig production is a vital source of livelihood for many smallholder farmers in Uganda, contributing significantly to food security and livelihoods. However, farmers face numerous challenges that hinder pig productivity and profitability. A critical aspect of disease control is the availability and accessibility of effective vaccines. This study aimed to identify barriers to the uptake of pig vaccines by practitioners and farmers in Uganda. We employed a systematic literature review with qualitative investigation, including focus group discussions (FGDs) and key informant interviews (KIIs) to characterise the pig vaccine supply chain, identify actors involved, barriers and challenges faced, and propose possible solutions. The study identified a supply chain involving multiple actors, including vaccine importers, wholesalers, retailers, and end users. Key barriers identified were policy weaknesses, incoordination, structural (poor staffing and funding of veterinary services), technical (knowledge gaps), logistical (limited infrastructure), and socio-economic (high costs).

**Contribution:**

This study highlights key barriers to farmers’ uptake of pig vaccines. It highlights a need for the Ugandan government to strengthen the regulation, control, and monitoring of pig vaccines. Given that policy and structural, technical, logistical, and socio-economic barriers exist at different nodes of the value chain, specific interventions are needed to address them. There is a need for capacity building of value chain actors – especially veterinary practitioners and farmers on the safe use and benefits of vaccines. The vaccine supply chain actors would benefit from increased investments in infrastructure, such as cold chain facilities, by public and private sector players. Future studies on the epidemiology of important diseases, vaccine efficacy, and socio-cultural barriers to vaccine uptake are recommended.

## Introduction

Pig production supports the livelihoods of many people in many developing countries as a source of food, income, and employment. In many low-income countries, such as Uganda, projections show that pork consumption will exceed production by 2030 (Erdaw 2023:8–10; Pica-Ciamarra et al. 2013:6–8). This may create a shortfall in pork supply, creating investment potential in the sector. In Eastern and Central Africa, Uganda is ranked second in pig production (behind Malawi), producing over 190 000 tonnes of pork annually, with a per capita pork consumption of 3.4 kg per year (Twine & Njehu 2020:7).

The Uganda 2021 National Livestock Census revealed an increase in the national pig population from 3.2 million in 2008 to 7.1 million in 2021, an increase of over 2.2 million households (UBOS 2021:105–108). Despite this, the pig sector has been neglected, with more emphasis placed on beef, dairy, and poultry value chains. Smallholder farmers raise most pigs in Uganda with minimal inputs in feeding, biosecurity, health, and welfare (Gertzell et al. 2021:461–462; Ikwap et al. 2014:39–47; Twine & Njehu 2020:28–32). However, the pig sector faces several challenges, including diseases, poor quality feeds, inferior genetics, high input costs, inadequate husbandry skills, and insufficient animal health technologies (Babigumira et al. 2023; Muhanguzi, Lutwama & Mwiine 2012). The success of veterinary services in terms of farmers’ access to critical inputs such as advisory services, drugs, and vaccines is largely determined by governance, structural, technical, and economic factors (Atherstone et al. 2019:6–10; Ilukor et al. 2015:17–26). While African Swine Fever remains a significant threat to the pig industry in Uganda, other endemic vaccine-preventable diseases, such as porcine reproductive and respiratory syndrome virus, porcine circovirus type 2 (PCV2), and porcine cysticercosis, occur (Kungu et al. 2019; Wilfred et al. 2018). In low-income settings, pig vaccines are scarce and inaccessible to most smallholder farmers (Donadeu et al. 2019:2–3). In Uganda, apart from a study evaluating the TSOL18 vaccine against *Taenia solium* cysticercosis (Nsadha et al. 2021), few studies have been conducted, indicating a lack of data on the benefits of vaccination for the swine industry. In Uganda, the use of vaccines in pigs is rarely practised, yet it is a proven effective tool for disease control used in other countries. Understanding barriers and challenges to their uptake among value chain actors is critical for guiding interventions. This study was conducted to characterise the vaccine supply chain from importation to use by farmers and to identify barriers to uptake to inform the design of interventions.

## Research methods and design

### Study design

In this study, we employed a cross-sectional design to identify barriers to vaccine uptake and use among pig farmers. A sequential exploratory qualitative study was done integrating two complementary data sources to identify key actors in Uganda’s pig vaccine supply chain, describe their roles, and profile the challenges they face. The approach included: (1) a systematic literature review and (2) key informant interviews (KIIs) and focus group discussions (FGDs) with relevant actors. In Uganda, farmers generally raise pigs in intensive, semi-intensive, or free-range systems, with minimal use of production inputs. Partly due to this, there is a high incidence of endemic diseases, resulting in low productivity and profitability.

### Phase 1: Systematic literature review

The review focused on literature published between 1995 and 2022. The starting point in 1995 was selected to capture developments following major constitutional and policy reforms in Uganda, which significantly shaped the structure and regulation of the veterinary and animal health sectors. It was assumed that the key actors and institutional frameworks along the pig vaccine supply chain would have remained relatively stable within this temporal window, making it an appropriate scope for trend analysis and actor mapping.

#### Search strategy and selection criteria

The literature review was performed using the Preferred Reporting Items for Systematic Reviews and Meta-Analyses Extension for Scoping Review (PRISMA-ScR) approach (Selcuk 2019:1–2). A comprehensive literature search was conducted across academic databases, including PubMed (NCBI, Bethesda, Maryland, United States) and Google Scholar (Mountain View, California, United States), as well as grey literature sources. The following search terms were used:

((pig OR pigs OR livestock) AND (vacc* OR drug) AND (Uganda) AND (supply chain OR value chain) AND (challenges)).

The search strategy was tailored for each source. To support systematic management of records and avoid duplication, Mendeley bibliographic software (Elsevier, London, United Kingdom) was used for reference management. For each source, the search date, years covered, and the number of retrieved records were documented at the time of import into Mendeley. For grey literature, we visited the websites of various institutions that engage in animal health in Uganda. These included the World Organization for Animal Health (WOAH), Food and Agriculture Organization of the United Nations (FAO), the African Union – Inter African Bureau for Animal Resources (AU-IBAR), International Livestock Research Institute (ILRI), the Global Alliance for Livestock Veterinary Medicines (GALVmed), Health for Animals, and pharmaceutical companies (Zoetis, ERAM).

#### Screening, inclusion, and exclusion criteria

A two-step screening process was employed: (1) title and abstract screening to remove irrelevant articles, and (2) full-text screening to assess the relevance of articles to the research objectives. Articles were considered eligible if they addressed the pig vaccine supply chain, policy context, and barriers or challenges in Uganda. Those excluded focused solely on human vaccines or other livestock other than swine. Copies of full articles were obtained from papers that matched the research question. Each article was independently reviewed by two researchers (Emmanuel Hasahya and Peter Oba) before inclusion. Discrepancies were resolved through consensus. The extracted data were entered into Excel, including: (1) author(s), year of publication, study location; (2) actors in the vaccine supply chain, (3) the interrelationships between the actors, (4) challenges faced, and (5) their proposed solutions.

### Phase 2: Qualitative data collection

#### Study locations

**FIGURE 1:**
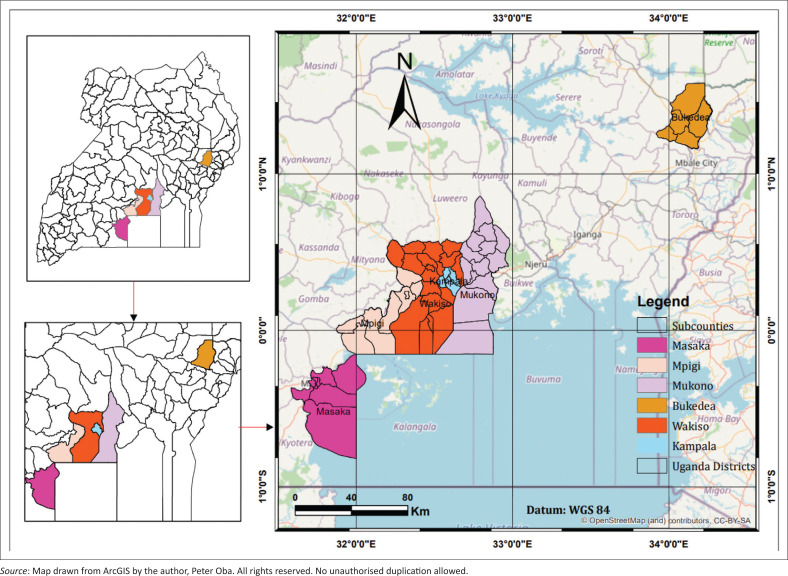
Map of Uganda showing study sites – Mukono, Kampala, Wakiso, Bukedea, Masaka, and Mpigi districts.

The study was conducted in Kampala, Mukono, Wakiso, Masaka, Bukedea, and Mpigi districts. Kampala was purposely selected because of a high concentration of vaccine importers and wholesalers or distributors. Mukono, Wakiso, and Mpigi districts (urban, semi-intensive) were purposively selected due to their relatively high pig population density, proximity to vaccine distributors, and the larger urban market for pork in Kampala (Kungu et al. 2017:1–2). Masaka and Bukedea districts were purposely selected to represent peri-urban (semi-intensive) and rural (free-range) pig production systems, respectively. Figure 1 shows a map of the study areas.

#### Key informant interviews and focus group discussions

Three (*n* = 3) key informant tools were developed specifically for each of the vaccine importers, wholesalers, and distributors. Key informant interviews’ tools were pretested in Mukono and revised before use. Guiding questions for the FGDs were prepared and used to guide discussions. Key informants (KIs) were interviewed (by Emmanuel Hasahya and Peter Oba) and included vaccine importers, wholesalers or distributors, retailers or veterinary vaccines and drug shops (VVDSs), and animal health workers. The KIs were interviewed to identify: (1) the vaccine types traded, (2) the sources and prices of vaccines, (3) vaccine buyers, (4) the volumes traded, (5) the challenges faced, and (6) suggestions on how to improve the vaccine supply chain. Each FGD involved stakeholders in the vaccine supply chain (vaccinators, vaccine and drug shop attendant, vaccine distributors and pig vaccine importers). For each FGD, there was a session facilitator and a notetaker who recorded responses following a checklist.

#### Data analysis

Data were summarised by drawing a map of a pig vaccine supply and delivery chain from importation to end users. Thematic analysis was used for data analysis. Raw interview data were transcribed and coded to identify recurring themes or concepts to elicit any similarities or differences that emerged. Based on a hypothetical scheme we generated and that was used in previous studies on adoption and uptake of animal vaccines in smallholder settings (Donadeu et al. 2019), we identified four key components of barriers, which include policy and regulatory, technical, structural, and logistical or infrastructural barriers. All participant responses were re-checked and verified to determine whether our coding scheme fit the data and whether a new coding scheme emerged. Given the limited number of players in the vaccine value chain, no attempts were made to examine differences between districts.

#### Supply chain mapping

A pig vaccine supply chain map was developed to visually represent the flow of vaccines, the interconnections among actors identified in the study, and the regulatory framework governing the supply chain. The map was constructed by synthesising data collected from the reviewed articles, KIIs, and FGDs. Actors mentioned by participants included importers, wholesalers or distributors, VVDSs, vaccinators (who double as animal health workers), and pig farmers. These were categorised by function and level. Relationships and flows, such as procurement, distribution, and service delivery, were mapped from descriptions provided by respondents and refined by comparing across stakeholder groups.

### Ethical considerations

This study received ethical approval from Uganda’s Vector Control Division Research Ethics Committee (VCD REC ref. no. UG-REC-018) and ILRI’s Institutional Review Ethics Committee (ref no. ILRI-IREC2022-41). Written informed consent was obtained from human participants who took part in the study. Information about research participants was anonymised, so no identifying information about participants is presented.

## Results

### Screening and selection of studies

**FIGURE 2:**
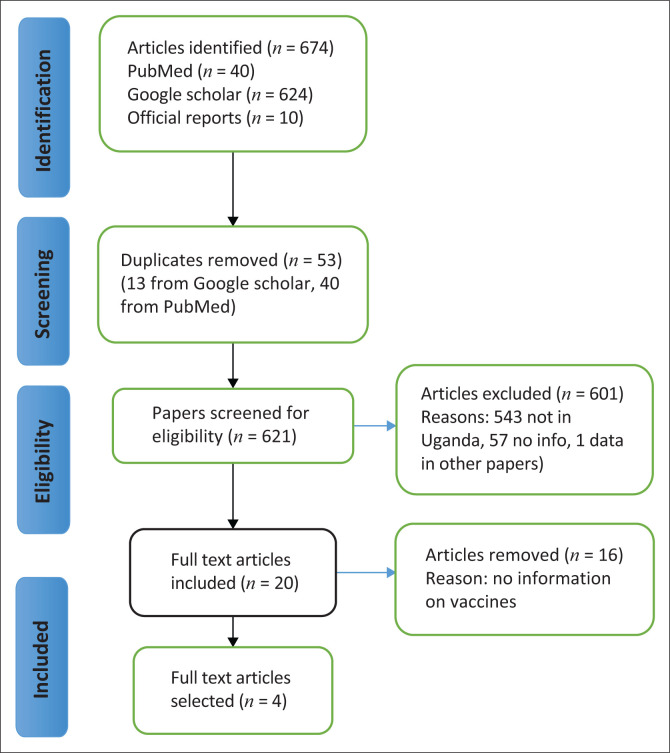
Preferred reporting items for systematic reviews and meta-analysis flow scheme used for identification, screening, and inclusion of articles for the systematic desk review.

The systematic literature search initially identified 674 articles, from which 53 duplicates were removed. After screening the titles and abstracts of the remaining 621 unique records, 601 were excluded as irrelevant. This left 20 articles for full-text review, of which only four met the eligibility criteria (Figure 2).

Additionally, no articles retrieved from grey literature sources were deemed eligible for inclusion. Data extracted from the retained articles are shown in Table 1.

**TABLE 1:** Summary of the four studies retained in the systematic literature review, showing location, actors, policies, and challenges identified.

Authors	Location	Stakeholders interviewed	Challenges identified
Arvidsson et al. (2022)	Nwoya district, Northern Uganda	Makerere University lecturers (4) Field veterinarians (7) Para-veterinarians (7) Farmers (224)	Understaffing and underfunding of the veterinary sector (poor funding) Difficulty for smallholders in accessing veterinary services Knowledge gaps among para-veterinary practitioners, (e.g. on the use of pig vaccines, handling pig diseases, etc.) Limited access to veterinary care and pharmaceuticals Limited understanding of socio-cultural barriers to uptake of vaccines by farmers
Ouma et al. (2021)	Bukedea, Masaka	Pig farmers (294)	The marketing system does not reward food safety; the focus is on carcass weight Poor results from previous vaccinations Expensive pig vaccines
Dione et al. (2021)	Lira, Mukono	Private veterinarians (16), drug stockists (8) Researchers (3) NDA (2) Senior Veterinary Inspector (1) District Veterinary Officers (2) District Production Officer (1) Distributor or wholesaler (1) Drug retailers (13) Veterinary practitioners (100)	Repackaging of products into smaller units at different nodes of the supply chain Weak enforcement of regulations Weak quality control and assurance mechanisms Reported cases of poor effectiveness of some drugs and vaccines by farmers Poor storage and handling by retailers or inappropriate use by farmers High transaction costs Farmers’ reluctance to adopt interventions
Ilukor et al. (2015)	Anonymised districts	MAAIF District local governments Opinion leaders and veterinarians (international development agencies and NGOs)	Staff absenteeism Poor funding and staffing of veterinary services Weak legislation Exclusion of technical staff from the decision-making process and policy incoherence

Note: Please see the full reference list of the article, Hasahya, E., Oba, P., Ouma, E.A., Doyle, R., Knight-Jones, T.J.D. & Dione, M., 2026, ‘Supply chains and delivery systems for pig vaccines in Uganda – Challenges and solutions’, *Onderstepoort Journal of Veterinary Research* 93(1), a2246. https://doi.org/10.4102/ojvr.v93i1.2246, for more information.

NDA, National Drug Authority; MAAIF, Ministry of Agriculture, Animal Industry and Fisheries; NGOs, non-government organisations.

Table 2 summarises key informants (KIs) and FGDs held, and the number of participants interviewed. In all, a total of 32 key informants (23 males, 9 females) were interviewed, and three FGDs (17 males and 10 females) were held.

**TABLE 2:** Summary of the pig vaccines supply chain actors interviewed by district.

District	Number of KIs interviews held				Number of FGDs held	Number of participants per FGD
	Practitioners (vaccinators)	Veterinary vaccines/drug shops (VVDSs)	Drug distributor	Pig vaccine importers		
Kampala	2	2	2	1	1	6
Mukono	8	1	-	-	0	-
Wakiso	8	-	-	-	0	-
Masaka	8	2	-	-	1	11
Mpigi	6	-	-	-	0	-
Bukedea	-	10	-	-	1	10

**Totals**	**32**	**15**	**2**	**1**	**3**	**27**

Note: Drug distributors and vaccine importers in other districts were not available.

VVDSs, veterinary drug shops; FGD, focus group discussion; KIs, key informants.

### Vaccine supply map and value chain actors involved

Results from KIIs showed that Zoetis™ is the sole importer of pig vaccines in Uganda. Upon importation, pig vaccines are supplied in refrigerated trucks to wholesalers. The wholesalers then supply retail veterinary vaccine/drug shops (VVDSs) across Kampala, Mukono, Wakiso, and Masaka. Retail VVDSs are either supervised by veterinarians or veterinary paraprofessionals. A few affluent farmers (especially large-scale) also directly procure vaccines from wholesalers, on the advice of their veterinarians, and can then store them on-site in refrigerators until use.

Retail VVDSs supply to (1) the pig vaccinators who may be private or government animal health officers (often in the business of pig artificial insemination, also offering vaccination for their clients), and (2) some affluent large-scale pig farmers (Figure 2). The pig vaccinators often transport the vaccines in plastic cool boxes stacked with ice packs. Large-scale pig farms employ veterinarians or para-veterinarians who advise and administer vaccinations.

In some cases, the retail VVDSs, owned by veterinarians and para-veterinarians, buy vaccines from one another, especially during periods of scarcity. Similarly, some animal health workers reported sharing vaccines, especially when one of them acquires a vial of 50 doses that they cannot use before it expires. Some practitioners reported being offered vaccines in reconstituted form, often as single-dose syringes from retailers. The farmers either buy the vaccines directly from VVDSs or invite veterinarians and para-veterinarians to administer them.

### Barriers to the uptake of pig vaccines

**FIGURE 3:**
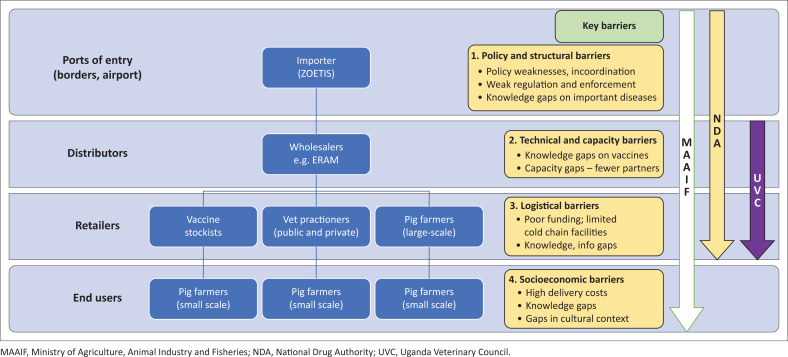
Mapping of Uganda’s pig vaccine supply chain, from port of entry to farmers and barriers at each node; large arrows on the right indicate the mandate of the Ministry of Agriculture, Animal Industry and Fisheries, National Drug Authority, and Uganda Veterinary Council.

Our study identified key barriers to vaccine uptake at each node of the value chain. We identified three regulatory agencies in the pig vaccines value chain. The Ministry of Agriculture, Animal Industry and Fisheries (MAAIF) is mandated to formulate and enforce policies on animal disease control and surveillance; the National Drug Authority (NDA) regulates importation, distribution and monitoring of veterinary drugs and vaccines, while the Uganda Veterinary Council (formally was Uganda Veterinary Board) licences veterinarians and para-veterinarians in public and private practice to ensure professional standards of practice are observed. Farmers (both large- and small-scale) demand veterinary inputs (drugs, vaccines, etc.) and services from veterinary practitioners and are the end users. Based on a systematic review and key informant interviews, we present a schematic representation of the pig vaccine supply chain, from importation to farmers and key barriers to uptake at each value chain node. This study identified key barriers to the uptake of pig vaccines that were related to policy and structural, technical, logistical, and socio-economic factors (Figure 3).

### Imported pig vaccine types traded, their characteristics, and unit prices

Results from the key informant interviews revealed that no pig vaccines were being manufactured in Uganda. Among the pig vaccines imported by Zoetis to Uganda are FarrowSure^®^ Plus B, LitterGuard and Fostera. FarrowSure^®^ Plus B is a multivalent vaccine that targets protection of breeding pigs from poor reproductive performance caused by porcine parvovirus (PPV), erysipelas caused by *Erysipelothrix rhusiopathiae*, and leptospirosis caused by *Leptospira bratislava, L. canicola, L. grippotyphosa, L. hardjo, L. icterohaemorrhagiae*, and *L. pomona* (Table 3).

**TABLE 3:** Pig vaccines marketed in Uganda and their characteristics.

Vaccine trade name	Target pathogen(s)	Target age groups	Dose and route of administration	Storage conditions	Immunity period
FarrowSure	Porcine parvovirus (PPV), *Erysipelothrix rhusiopathiae*, and *Leptospira spp* (*L. bratislava, L. canicola, L. grippotyphosa, L. hardjo, L. icterohaemorrhagiae*, and *L. pomona bacterin*)	Sows and gilts 2–4 weeks prior to breeding	5 mL intramuscular	Store at 2 °C – 7 °C.	26 weeks
LitterGuard	*Escherichia coli* enterotoxigenic strains having the K99, K88, 987P, or F41 adherence factors	Pregnant sows and gilts	2 mL intramuscular or Subcutaneous	Store at 2 °C – 7 °C.	2 weeks before farrowing
Fostera	Porcine circovirus type 2 (PCV2) and *Mycoplasma hyopneumoniae*	3-week-old piglets	2 mL dose intramuscular	Store in the dark at 2 °C – 7 °C.	23 weeks

The LitterGuard^®^ vaccine is a bacterin prepared from chemically inactivated strains of *Escherichia coli*, used to vaccinate healthy pregnant sows and gilts to enable passive vertical transfer of protective maternal antibodies to neonates against neonatal diarrhoea caused by enterotoxigenic strains of *E. coli* with the K99, K88, 987P, or F41 adherence factors. The vaccine is administered to healthy pregnant sows or gilts in 2 doses, 3 weeks apart during the last half of pregnancy, with the second dose given at least 2 weeks before farrowing. The sows can be revaccinated with a single dose at least 2 weeks before each subsequent farrowing.

The Fostera™ PCV MH vaccine is used for healthy pigs 3 weeks of age or older and provides immunity for 23 weeks. It is used to prevent viremia and lymphoid depletion caused by PCV2 and *Mycoplasma hyopneumoniae*-induced pneumonia.

Typically, distributors apply a 15% transportation charge from the manufacturer to Kampala, with a 30% mark-up profit. Further downstream, drug stockists in districts include an additional 30% markup as their profit. The pig farmer is charged between UGX 15 000.00 – UGX 30 000.00 ($4.00 –$8.00) per dose, depending on how far the farm is from the vaccinator (Online Appendix 1 – supplementary Table 1). Table 3 summarises vaccine types, target pathogens, pig age groups, doses, routes of administration, and storage conditions.

Table 4 summarises key barriers to the uptake of pig vaccines in selected districts in Uganda identified by different study methods. Results show that while the systematic literature review (SLR) identified weaknesses in livestock sector policies, vaccine quality assurance, and reporting mechanisms, KIIs and FGDs revealed actor incoordination (between public and private sector players), weaknesses in institutional arrangements, and knowledge gaps among value chain actors for the delivery of vaccines to end users. All three studies consistently identified knowledge and information gaps as key technical and capacity barriers to vaccine uptake among different value chain actors. Regarding the logistical barriers, a lack of or limited access to vaccine cold chain facilities was highlighted by both the SLR and KI interviews. At the end-user node of the vaccines value chain, the SLR identified a lack of understanding of the socio-cultural barriers to vaccine uptake, while the KIIs and FGDs revealed high delivery costs, a shortage of partners to deliver vaccines to farmers, and misinformation about vaccines.

**TABLE 4:** Summary of key barriers to the uptake of pig vaccines by the three methods used.

Key barriers to vaccine uptake	Systematic literature review	Key informant interviews	Focus group discussions
Policy and structural barriers	Policy weaknesses and incoherence, e.g. failure to subsidise vaccines against pig diseases Lack of regulation and weak policy enforcement Weak quality assurance mechanisms	Knowledge gaps on important pig diseases Lack of coordination between public and private veterinary practitioners	Knowledge gaps among farmers on the use and economic benefits of vaccines Weak institutional arrangements among farmers
Technical and capacity barriers	Poor staffing of veterinary services Knowledge gaps on diseases among paraprofessionals Lack of knowledge on epidemiology of endemic diseases and strains for which vaccines are efficacious Lack of field monitoring on vaccines performance	Limited knowledge among practitioners on the safe use of vaccines Lack of field monitoring of vaccines performance	Knowledge and information gaps on the safety and cost benefits of vaccines Lack of mechanisms to report vaccine adverse effects
Logistical or infrastructural barriers	Limited cold chain facilities to maintain vaccine quality and safety	Limited cold chain facilities to maintain vaccine quality and safety in the field	-
Socio-economic or cultural barriers	Poor staffing and funding of veterinary services Limited understanding of socio-cultural barriers to uptake of vaccines for marginalised groups	Insufficient partners (practitioners) to deliver vaccines to farmers Poor facilitation to reach farmers Remoteness of farmers Social and gender related barriers	High vaccine costs Misinformation about vaccines is causing fear among farmers to use vaccines

## Discussion

Mapping the pig vaccine supply chain is a key step to aid in identifying institutional and structural gaps and weaknesses, helping overcome challenges faced by various actors. This will enable the private sector, development partners, and government policy makers to plan for and regulate the importation and distribution of pig vaccines. In this paper, we identify the actors involved in the pig vaccines supply chain and describe their roles, the institutional structures under which they operate, and the challenges they face. This study identifies four categories of barriers to the uptake of pig vaccines in Uganda: Policy and structural, technical and capacity, logistical, and socio-economic barriers.

### Policy and structural barriers

Our findings reveal barriers in national livestock sector policy, poor enforcement, and weak institutional structures that hinder the uptake of pig vaccines. Unlike vaccines for priority livestock diseases, pig vaccines are not treated as a public good, leaving their distribution to a private monopoly. Under the current animal health policy, the government of Uganda is mandated to control five priority livestock diseases – *peste des petits ruminants (PPR)*, contagious bovine pleuropneumonia, foot and mouth disease, rabies, and anthrax (MAAIF 2022:11). While farmers are required to contribute a fraction of vaccination costs, their costs are subsidised by the government (MAAIF 2022:11). Such policy incoherence makes it difficult to enforce any regulations, since public actors alike rely on a single importer for pig vaccines.

Findings from the systematic review and key informant interviews revealed that all vaccine distributors and most veterinary practitioners involved in the pig vaccines trade were private dealers. Such arrangements make it difficult to regulate their field practice. At the import node, no structures or mechanisms were established to obtain feedback on vaccine performance from practitioners and farmers. KIIs revealed that there was little to no coordination between public and private sector players, and that weak institutional arrangements existed to ensure collective demand and bargaining for pig vaccines. While NDA is legally mandated to monitor vaccine adverse effects, institutional structures were weak, poorly facilitated, or nonfunctional. Results from the systematic review and KIIs reported that some veterinary drug shop owners engaged in malpractices such as selling vaccines directly to pig farmers. While their prices were lower than those of veterinary practitioners, such malpractice is risky, as farmers lack the knowledge to safely transport and administer vaccines to pigs. Such malpractice occurs only in situations where regulations are weakly enforced. The consequences of such poor vaccine handling practices have been observed among poultry farmers, where vaccine adoption rates are higher than in pigs. A policy change that requires enhanced coordination between public and private vaccine value chain actors could improve regulatory oversight and monitoring of vaccine performance in the field.

### Technical and capacity barriers

Technical and capacity barriers were identified across all vaccine value chain nodes in this study. At the importation node, importers lack knowledge of the epidemiology of endemic pig diseases, which is needed to inform their choices of vaccine types to import. Such knowledge gaps were highlighted by both KIIs and FGDs. While previous studies demonstrated the occurrence of vaccinatable diseases such as porcine circovirus type 2 (PCV2), *Mycoplasma pneumoniae* (*M. hyo*), and porcine reproductive and respiratory syndrome virus (Oba 2023; Wilfred et al. 2018). Such information did not inform the importer’s vaccine choices but was instead based on demand. At the wholesale/retail node, a systematic literature review identified knowledge gaps among practitioners and farmers on vaccines and pig diseases (Table 4). For example, KIIs revealed that some practitioners used incorrect routes for vaccine administration, under-dosed pigs or misused vaccines (e.g. using vaccines not indicated for the ages or physiological state of pigs), which calls for refresher training, licensing and stronger regulation. As stated, ‘Veterinarians don’t know about pig vaccines; sometimes some of them use LitterGuard to vaccinate older pigs’ (Key informant from Masaka). A new regulation by the Uganda Veterinary Council (UVC) requiring veterinarians and para-veterinarians to undergo further continuous professional development to obtain an annual practice licence may help close this knowledge gap.

In this study, most FGD respondents were unaware of the availability and benefits of pig vaccines. As one FGD observed, ‘As farmers, we do not know how those vaccines can benefit our pigs’ (FGD participant, Mukono district). Among those who attempted to vaccinate their pigs, some reported cases of abortions. Such negative experiences discourage other farmers. In Kenya and Uganda, high direct and indirect costs, fear of vaccine side effects, and limited information have been identified as key barriers to uptake of Rift Valley Fever vaccines (Mutua et al. 2019:7–9). Partly due to a lack of subsidies for pig vaccines, farmers are not supported in accessing the necessary knowledge and information (e.g. through training) to make informed choices. At the import node, the importer has limited knowledge of the epidemiology of endemic pig diseases, which is needed to inform their vaccine choices for import. Such knowledge gaps were highlighted by both the KIIs and FGDs. To address technical and capacity barriers to vaccine uptake, there is a need to build the capacity among various actors (Bugeza et al. 2017; Donadeu et al. 2019).

### Logistical or infrastructural barriers

Logistical infrastructure for safe vaccine handling, such as fridges, cool boxes, and reliable electricity supply for veterinary practitioners, was found to be inadequate or lacking. This not only disincentivises vaccine uptake but also increases operational costs for practitioners. The KIIs and systematic review observed that during power cuts, some dealers continue to sell vaccines to practitioners and farmers without regard to their safety and efficacy. In addition, limited access to fridges and cool boxes by private veterinary practitioners raises concerns about vaccine viability and efficacy, which likely explains the poor results experienced by some farmers. This problem was noted to discourage vaccine uptake, even among farmers who would otherwise be willing to adopt vaccination.

Previous studies emphasised the importance of involving local veterinary officials and support infrastructure in the eradication of rinderpest (Acosta, Hendrickx & Mckune 2019:5–6; Mariner et al. 2012:1309–1312). Enhancing the cooperation and coordination between public and private veterinary practitioners through the sharing of logistics, such as fridges and cool boxes, could help reduce the costs of vaccine delivery and improve uptake by farmers, as proposed in a previous study (Ilukor et al. 2015). A vaccine delivery model in which few veterinarians leveraged on large numbers of community animal health workers to deliver vaccines to remote areas was effective in rinderpest eradication (Mariner et al. 2012:1309–12). Designing effective vaccination campaigns requires a good understanding of the structure of veterinary services, technical competencies and logistical capacities (Ayebazibwe et al. 2022:71–73; Ilukor, Birner & Nantima 2025:18–20).

### Socio-cultural barriers

The use of vaccines in remote areas where most smallholder farmers live was virtually non-existent because there were no vaccine distribution outlets. While the SLR highlighted gaps in understanding of the economic and socio-cultural contexts in which vaccines are to be applied, KIIs and FGDs revealed high transaction costs, a lack of partners, and misinformation about vaccines. For example, farmers need to know the benefits of vaccines to encourage uptake, yet they lacked this knowledge and information. At the distribution node, KIIs revealed fewer practitioners in the field and the remoteness of farmers. A lack of understanding of the socio-cultural context of vaccine uptake was highlighted by the systematic literature review, while the FGDs reported misinformation about vaccines. Key informant respondents reported high import taxes levied on veterinary drugs and vaccines, which increased downstream costs and ultimately discouraged vaccine uptake among end users. This finding agreed with a previous study (Ilukor et al. 2025:18–20). It was reported that high transaction costs for distribution and inadequate supply are a major hindrance to vaccine use by farmers (Ayebazibwe et al. 2022). Socio-cultural factors such as ethnicity, gender, and remote location were cited as markers of exclusion and marginalisation in accessing livestock vaccines in the Karamoja region (Arvidsson et al. 2022; Serra et al. 2022). The involvement of women and youths through gender-sensitive and inclusive delivery models has been observed to improve vaccine access and reduce transaction costs (Mutua et al. 2019:7–8). When planning and designing vaccination campaigns, it is critical to consider socio-cultural and gender-responsive approaches that increase women’s participation in the vaccine value chain (Njiru et al. 2024:9–10; Serra et al. 2022:1–2). Such strategies have been shown to improve vaccine coverage in hard-to-reach areas and foster more sustainable and equitable animal health systems.

### Limitations of the study

This study was limited to pig vaccine value chain actors, mainly in the central region of Uganda. Because of the small number of actors involved and interviewed per district, this limited the assessment of any differences in vaccine uptake levels between districts. The systematic literature review was based on only a few published studies, which limited the generalisability of its findings. However, overall, this study highlights key barriers to the uptake of pig vaccines and presents a fair picture of the pig vaccines supply chain in Uganda, as the studied districts are where major pig vaccines value chain actors are based.

## Conclusion

### Recommendations

This survey describes the structure, barriers, and challenges faced by various actors in Uganda’s pig vaccine supply chain. It highlights an urgent need for the government of Uganda to strengthen the regulation and control of pig vaccines. Given that policy, structural, technical, logistical, and socio-economic barriers exist across the different nodes of the value chain, specific interventions are needed to address them. There is a need for policy and structural review, capacity building of value chain actors – especially veterinary practitioners and farmers on the safe use and benefits of vaccines, and improving monitoring of vaccines. The vaccine supply chain actors would benefit from increased investments in infrastructure, such as cold chain facilities, by public- and private-sector players. Future studies to understand the epidemiology of important vaccine-preventable pathogens and on vaccine efficacy are recommended.
